# Quercetin Attenuates Lactate Production and Extracellular Matrix Secretion in Keratoconus

**DOI:** 10.1038/srep09003

**Published:** 2015-03-11

**Authors:** T. B. McKay, D. Lyon, A. Sarker-Nag, S. Priyadarsini, J. M. Asara, D. Karamichos

**Affiliations:** 1 Department of Cell Biology, University of Oklahoma Health Sciences Center, Oklahoma City, OK 73104, USA; 2 Department of Ophthalmology/Dean McGee Eye Institute, University of Oklahoma Health Sciences Center, Oklahoma City, OK 73104, USA; 3 Division of Signal Transduction, Beth Israel Deaconess Medical Center and Department of Medicine, Harvard Medical School, Boston, Massachusetts, USA

## Abstract

Keratoconus(KC) is an ecstatic corneal disease leading to corneal-thinning and the formation of a cone-like cornea. Elevated lactate levels, increased oxidative stress, and myofibroblast formation have all been previously reported. In the current study, we assess the role of Quercetin on collagen secretion and myofibroblast formation in KC *in vitro*. Human corneal fibroblasts(HCFs) and human keratoconus cells(HKCs) were treated with a stable Vitamin C derivative and cultured for 4 weeks, stimulating formation of a self-assembled extracellular matrix. All samples were analyzed using Western blots and targeted tandem mass spectrometry. Our data showed that Quercetin significantly down regulates myofibroblast differentiation and fibrotic markers, such as α-smooth muscle actin (α-SMA) and Collagen III (Col III), in both HCFs and HKCs. Collagen III secretion was reduced 80% in both HCFs and HKCs following Quercetin treatment. Furthermore, Quercetin reduced lactate production by HKCs to normal HCF levels. Quercetin down regulated TGF-βR2 and TGF-β2 expression in HKCs suggesting a significant link to the TGF-β pathway. These results assert that Quercetin is a key regulator of fibrotic markers and ECM assembly by modulating cellular metabolism and TGF-β signaling. Our study suggests that Quercetin is a potential therapeutic for treatment of corneal dystrophies, such as KC.

According to the National Eye Institute, over 155,000 Americans are affected by Keratoconus (KC) with 20% of those reaching severe stages requiring corneal transplantation^1^. KC results in the formation of a cone-like cornea and impaired vision due to compromised corneal integrity, degeneration of the basal epithelial cells, and breakage of Bowman's membrane^2^,^3^,^4^. Current treatments for mild to advanced KC patients include rigid gas permeable lenses (RGPs) and corneal crosslinking^5^,^6^. These treatments serve to slow and, in some cases, halt the progression of the disease by increasing collagen fibril linkages within the cornea, thereby preventing extreme curvature. Corneal transplantation is currently the only available treatment for severe-stage KC patients.

In the normal eye, the corneal stroma consists of the resident cells termed keratocytes and the assembled extracellular matrix (ECM). The corneal stroma accounts for up to 90% of total corneal thickness and provide the structural basis for the anterior segment of the eye^7^. Any disruptions in collagen architecture, collagen mechanical properties, and collagen-linking enzyme activity can lead to altered ECM assembly and a defective corneal structure, as observed in KC.

One of the main characteristics of KC is the significant loss of corneal ECM^8^,^9^. Unfortunately, to-date, there are no treatments available to reverse or recover ECM loss and generate a more normal stroma-like cornea. A fundamental hurdle in developing therapeutics to treat KC is largely due to the lack of a clear mechanism of the molecular basis for the development and progression of this disease, as well as the absence of animal models. Studies involving KC patients have focused on the analysis of tear and serum samples^10^,^11^,^12^,^13^,^14^, while the majority of studies in vitro are utilizing 2D conventional cell culture models. We have previously developed a 3D in vitro model^15^ showing that primary human keratoconus cells (HKCs) are terminally differentiated to myofibroblasts and are unable to secrete comparable amounts of ECM relative to normal human corneal fibroblasts (HCFs)^16^. We have also reported significant dysregulation of key metabolites associated with glycolysis, citric acid cycle, and oxidative stress in both 2D and 3D KC in vitro models^17^. Other studies have also associated KC with mutations in mitochondrial genes^18^, elevated mitochondrial damage^19^, increased sensitivity to oxidative stress^20^,^21^, and increased apoptosis^22^.

Cellular metabolism is critical for maintaining homeostasis in the tissue microenvironment by determining cytoplasmic functions within the cell itself, as well as influencing the composition of the extracellular niche. In the cornea, stromal keratocytes are dispersed within the ECM and are directly affected by changes in metabolite flux and ECM structure. Metabolite characterization has been used by our group^17^ and others^23^,^24^ to investigate molecular characteristics of disease, including KC. We have previously reported alterations in the key metabolic pathway utilized by HKCs in primary energy production favoring aerobic glycolysis and characterized by excess lactate production^17^. Among other metabolites, lactate is a metabolite known to be highly regulated both in HKCs^17^ and KC corneal buttons^25^. Lactate has also been implicated in corneal edema *in vivo* promoting fibrosis during wound healing via activation of transforming growth factor- β (TGF-β) signaling^26^,^27^. In this study we examined the effect of a lactate transporter inhibitor, known as Quercetin, on ECM assembly, fibrosis, and cellular metabolism in KC.

Quercetin is a naturally produced flavonoid (Figure 1) that has been reported to exhibit antifibrotic and antioxidant properties in primary orbital fibroblasts and hepatocytes^28^,^29^,^30^,^31^,^32^. Several studies have identified Quercetin as a potent inhibitor of monocarboxylate transporter 1 (MCT1), which is a plasma membrane transporter of lactate, pyruvate, and ketone bodies^33^,^34^,^35^. MCT1 is ubiquitously expressed throughout the body, including the cornea^36^. However, to the authors' knowledge, there is no report of the effects of Quercetin on KC *in vitro* or *in vivo*. The purpose of this study was to evaluate the effect of Quercetin on HKCs using our 3D in vitro model^15^. In addition, we examined the effects of Quercetin on cellular metabolism and fibrosis using a variety of techniques to confirm our findings. Our results show that Quercetin significantly decreases collagen secretion and the expression of pro-fibrotic molecules via TGF-β signaling pathway. Overall, we have identified Quercetin as a potential novel therapeutic to reduce scarring and attenuate oxidative stress regulatory signals involved in the pathophysiology of KC.

## Results

### Lactate stimulation: Fibrotic effects

**Figure 2:**
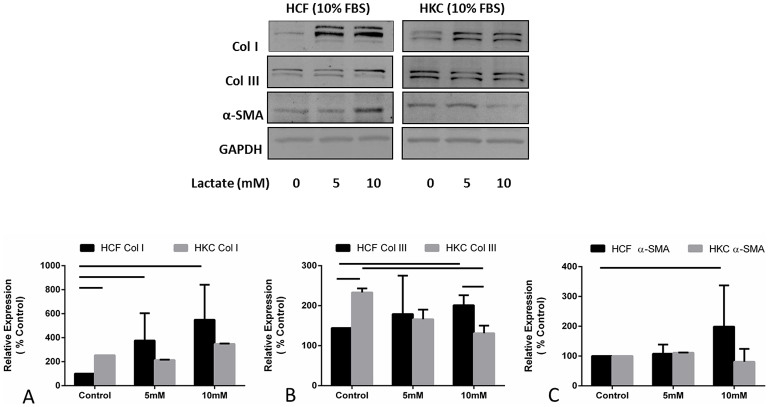
Quantification of western blots of HCF and HKC cell lysates following four week treatment with increasing concentrations of lactate. (A) Col I expression was significantly up regulated (p < 0.05) in HCFs following 5 and 10 mM exogenous lactate stimulation, (B) Col III was significantly up regulated (p < 0.05) in HCFs following 10 mM exogenous lactate stimulation. Col III expression was significantly higher at HKC-Control compared to HCF-Control (p < 0.05), and (C) α-SMA was significantly up regulated in HCFs (p < 0.05) following 10 mM exogenous lactate stimulation. All gels have been run under the same experimental conditions. n = 3, error bars represent (SEM).

Lactate has been linked to oxidative stress^37^,^38^ and cornea edema^26^. We have also previously reported its link to KC disease in vitro^17^. Here, we tested the effects of exogenous lactate stimulation at physiological pH within the cornea on both HCFs and HKCs. Figure 2 shows regulation of Col I (Figure 2A), Col III (Figure 2B), and α-SMA (Figure 2C) on HCFs and HKCs at 0, 5, and 10mM lactate concentrations. Col I expression was significantly up regulated in HCFs following 5 (Figure 2A; p < 0.05) and 10 mM (Figure 2A; p < 0.05) lactate stimulation. However, no significant difference was seen between 5 and 10 mM concentrations. Conversely, HKCs showed no significant up-regulation of Col I upon lactate stimulation (Figure 2A). Col III expression was significantly up-regulated in HCFs following lactate stimulation at 10 mM (100%; Figures 2B; p < 0.05). Interestingly, Control HKCs showed approximately 125% higher expression of Col III when compared to HCFs (p < 0.05; Figure 2B), while there was almost a 2-fold lower expression of Col III upon 10 mM lactate stimulation (p < 0.05; Figure 2B). Lactate stimulation had the biggest impact on Col III expression in HKCs with a 100% reduction seen between HKCs Control and HKCs at 10 mM lactate. α-SMA expression was only modulated in HCFs upon 10 mM lactate stimulation, where a slight but significant up regulation was noted (Figure 2C; p < 0.05) These results support earlier published data^17^ that HKCs have a fibrotic phenotype and suggest a potential role for excessive lactate production in promoting scarring in fibrotic diseases.

### Quercetin decreases collagen secretion into media

**Figure 3:**
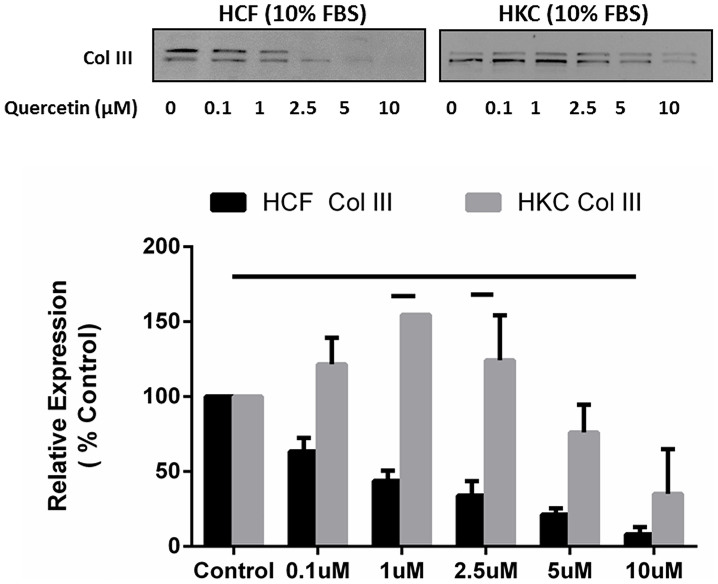
Quantification of Collagen III secretion into the culture media by HCFs and HKCs. (A) Representative western blots of Col III regulation is shown with increasing doses of Quercetin (0, 0.1, 1, 2.5, 5, and 10μM). (B) Quantification of western blots for both HCFs and HKCs is shown. Quercetin stimulation of 10μM led to significant down regulation (p < 0.05) of Col III in HCFs media. HKCs showed higher Col III expression, when compared to HCFs, at 1 and 2.5 μM of Quercetin. All gels have been run under the same experimental conditions. n = 3, error bars represent SEM.

In order to identify the effect of Quercetin on ECM secretion in HCFs and HKCs, we quantified the Col III amounts released into the culture media (Figure 3). We measured by Col III secretion (Figure 3) into the culture media following incremental Quercetin concentrations (0.1, 1, 2.5, 5, and 10μM). Figure 3 shows a linear decrease in Col III expression in 20% increments by HCFs (Figure 3; p < 0.05). HKCs showed elevated Col III expression at low Quercetin doses (0–1μM; p < 0.05) and reduced Col III levels (90%) at higher doses (5–10μM) of Quercetin treatment (Figure 3; p < 0.05).

Col III levels were significantly higher in HKCs when compared to HCFs at 1 and 2.5μM of Quercetin (Figure 3; p < 0.05). No toxicity of Quercetin was observed in our in vitro model. Several studies^39^,^40^ have used similar concentrations up to 50–100μM Quercetin in vitro.

### Quercetin stimulation: Anti-fibrotic effects

**Figure 4:**
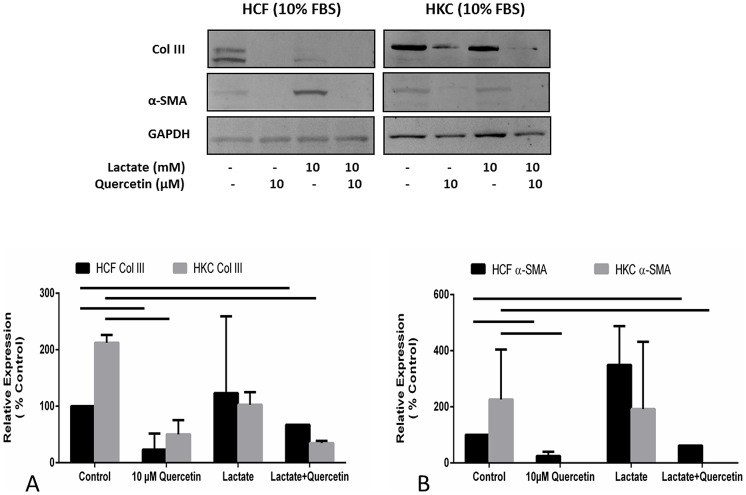
Quantification of western blots of HCF and HKC cell lysates following four week treatment with or without Quercetin and/or Lactate treatment. (A) Col III expression was significantly down regulated (p < 0.05) in both HCFs and HKCs following 10μM Quercetin stimulation. Lactate itself did not have a significant effect on Col III levels in either cell type, however combination of Lactate and Quercetin led to significant down regulation of Col III (p < 0.05). (B) α-SMA expression followed similar pattern to Col III. It was significantly down regulated in both HCFs and HKCs following 10μM Quercetin stimulation (p < 0.05) and in combination of Lactate and Quercetin. Lactate itself did not have a significant effect on α-SMA levels in either cell type. All gels have been run under the same experimental conditions. n = 3, error bars represent SEM.

Since we identified that Quercetin inhibited Col III secretion, we sought to investigate if expression of Col III was inhibited independent of secretion and reflective of general inhibition of myofibroblast differentiation. We measured Col III and α-SMA expression in the presence and absence of Quercetin (Figure 4). Figure 4 shows that 10μM Quercetin treatment significantly inhibited expression of Col III (Figure 4A; p < 0.05) and α-SMA (Figure 4B; p < 0.05) in the presence and absence of lactate in both HCFs and HKCs. The reduction in Col III and α-SMA expression following Quercetin treatment suggests that Quercetin is a potential anti-fibrotic that may be useful in the treatment of fibrotic diseases.

### Quercetin alters metabolism in HCFs and HKCs

**Figure 5:**
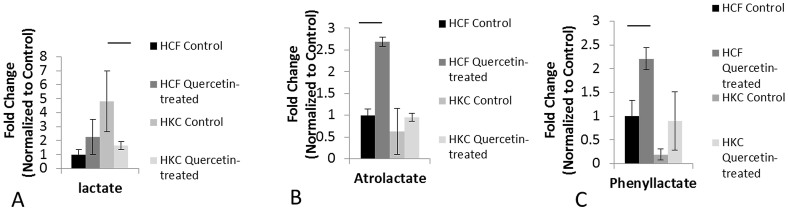
Quantified metabolomics data showing significant differences in lactate metabolite and lactate-derivatives: (A) Lactate metabolite showing significant down regulation of expression in HKCs following Quercetin stimulation (p < 0.05), (B) Atrolactate showing significant up regulation of expression in HCFs following Quercetin stimulation (p < 0.05), and (C) Phenyllactate showing significant up regulation (p < 0.05) of expression in HCFs following Quercetin stimulation.n = 3, error bars represent SEM.

Since Quercetin is a known inhibitor of the MCT1 lactate transporters^33^,^41^, we investigated whether Quercetin can affect intracellular lactate production in vitro by activating a negative feedback loop which would thereby decrease secreted lactate concentrations. We used a metabolomics analysis^23^,^24^ to identify variations in key metabolites in HCFs and HKCs following Quercetin treatment. We observed significant changes in specific metabolite concentrations, such as lactate and lactate-derived metabolites (Figure 5). Figure 5A shows a 5-fold increase in lactate production by untreated HKCs compared to HCFs. Quercetin treatment caused a dramatic decrease in lactate production by HKCs (Figure 5A; 2-fold, p < 0.03) and thereby restored lactate levels to the normal HCF levels. Interestingly, identical regulation was seen with two other key metabolites that are generated by phenylalanine and lactate metabolism, atrolactate (Figure 5B) and phenyllactate (Figure 5C). Figures 5B and 5C indicate that Quercetin did not cause a significant increase of these two metabolites in HKCs compared to HCFs, which showed a significant increase (2.5-fold, p < 0.05; and 2-fold, p < 0.05, respectively). Our data suggest that Quercetin is directly inhibiting the production of the excess lactate by HKCs rather than increasing lactate-derived metabolites.

### Quercetin: link to TGF-β pathway

**Figure 6:**
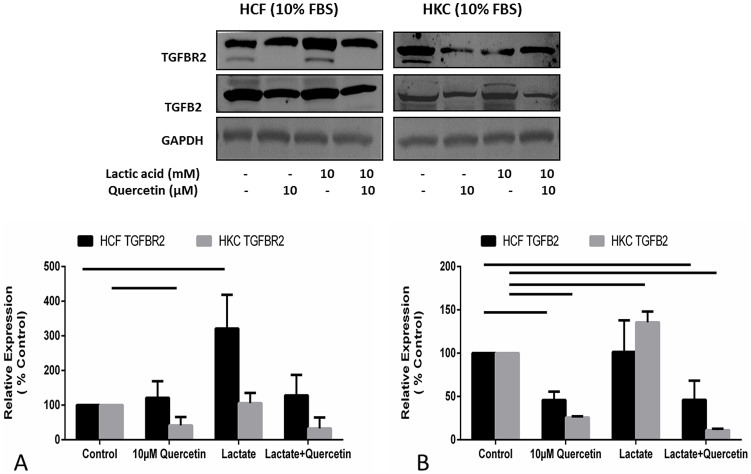
Quantification of western blots of HCF and HKC cell lysates following four week treatment with or without Quercetin and/or Lactate treatment. (A) TGF-βRII expression was significantly up regulated in HCFs following lactate stimulation (p < 0.05) and down regulated in HKCs following Quercetin stimulation (p < 0.05), (B) TGF-β2 expression was significantly up regulated in HKCs following Lactate stimulation (p < 0.05) where HCFs showed no differences. Quercetin stimulation led to significant down regulation of TGF-β2 expression in both HCFs and HKCs. All gels have been run under the same experimental conditions. n = 3, error bars represent SEM.

Previous studies by us^15^,^17^ and others^25^,^42^,^43^,^44^,^45^ have indicated a link between KC pathophysiology and the TGF-β pathway. In order to determine if Quercetin inhibited terminal myofibroblast differentiation in a TGF-β dependent manner, we investigated changes in the TGF-β pathway. TGF-β2 and TGF-β receptor II (TGF-βRII) levels were highly modulated. In HCFs, we identified a substantial increase in TGF-βRII expression (Figure 6A; 4-fold; p < 0.05) following 10 mM lactate treatment but no change in TGF-β2 (Figure 6B). HKCs on the other hand showed no significant change in TGF-βRII expression (Figure 6A) but significantly up regulated TGF-β2 (Figure 6B; p < 0.05). In agreement with our findings described above, Quercetin significantly decreased TGF-β2 and TGF-βRII expression in HCFs and HKCs even in the presence of excess lactate (Figure 6A and 6B). This data overall indicated a plausible link between elevated TGF-β activation and increased Col III and α-SMA expression. These results suggest a possible role of aberrant TGF-β signaling in altered ECM assembly by HKCs and the potential use of Quercetin to attenuate pro-fibrotic signaling.

## Discussion

KC is one of the most common corneal dystrophies that can lead to severe visual impairment^46^. KC is characterized by a bilateral, non-inflammatory progressive corneal ectasia that can lead to severe visual impairment once severe stages are reached^47^,^48^. To date, there is no therapeutic target available for the prevention of this disease, and the pathogenesis of KC remains unknown. In addition, there is no animal model available that can be used for studying the progression of the disease and promoting the development of novel treatments. The aim of this study was to identify, describe, and characterize the potential of Quercetin as a novel therapeutic to treat KC.

Quercetin is a common flavonol found in vegetables and fruits and is abundant in human diets. Studies have investigated Quercetin as an antioxidant^39^,^49^, anti-angiogenic^50^,^51^, neuroprotective^52^,^53^, and anti-apoptotic agent^54^,^55^. In ocular studies, Quercetin has been found to be efficacious in cataract prevention^56^, retinal angiogenesis^57^, and oxidative damage in retinal pigment epithelium cells^58^,^59^. Surprisingly, the number of published studies using Quercetin for corneal dystrophies is lacking.

In this study, we have identified Quercetin as a potent inhibitor of lactate-induced fibrosis in KC. Using a variety of established techniques, we have shown that Quercetin attenuates collagen secretion and terminal differentiation to the fibrotic phenotype observed in HKCs. Considering its antioxidant properties, as previously reported in the retina^58^,^59^, we hypothesized that Quercetin may target cellular metabolism in both HCFs and HKCs. Our data is in agreement with our previous observation^17^ that HKCs have altered cellular metabolism, including elevated lactate production compared to HCFs. Furthermore, the results shown here suggest that Quercetin modulates fibrotic signaling in HKCs by altering cellular metabolism and reducing lactate production. Analogs of lactate, including atrolactate and phenyllactate, which are primarily formed from lactate and phenylalanine metabolism, increased significantly in HCFs following Quercetin treatment, which suggests that Quercetin inhibits transport of any lactate produced by normal HCFs resulting in accumulation of cytoplasmic lactate metabolites. The lack of increase in both atrolactate and phenyllactate in HKCs suggests that Quercetin directly inhibits elevated lactate production by these cells. It is therefore plausible to postulate that the HKC phenotype may be corrected by altering metabolism in order to promote a normal ECM assembly. This hypothesis is supported by previous studies showing the potential importance of metabolism in KC disease^17^,^60^.

The role of cellular metabolism in regulating corneal fibrosis is not yet understood. Corneal fibrosis is a common feature associated with the KC pathology and is also a major clinical problem with currently only one treatment option: corneal transplantation. We have shown evidence here that Quercetin is a potential novel inhibitor of anti-fibrotic signaling and posit its potential for attenuating scar formation in KC and other diseases associated with fibrosis. As previously reported by us, HKCs are terminally differentiated to myofibroblasts with elevated α-SMA and Col III expression^17^. In the current study, we advance our knowledge by linking key fibrotic markers to elevated lactate levels and demonstrate how these may be responsible for promoting the HKC phenotype and altered cellular state. Based on our findings, excess exogenous lactate can induce HCFs to undergo terminal differentiation to a fibrotic phenotype similar to that observed in HKCs with fibrotic characteristics.

While our long term aim is to identify and completely characterize the cellular mechanisms in which HKCs are dysfunctional, we believe Quercetin can play a major role in modulating the altered cellular state to promote normality. Previous studies have identified variations in TGF-β signaling associated with KC^25^,^42^,^44^. We have shown in this study that Quercetin down regulates expression of both TGF-βRII and TGF-β2, which provides evidence of direct inhibition by Quercetin of pro-fibrotic markers promoted by TGF-β signaling. Collectively, our data show that Quercetin is a potent inhibitor of key fibrotic markers and a strong metabolic regulator in KC and highlight its potential as a therapeutic in the treatment of corneal scarring associated with KC.

## Methods

### Cell isolation and treatment

Both HCFs and HKCs were prepared as previously described^15^. HCFs were isolated from normal human corneas obtained from NDRI (National Disease Research Interchange; Philadelphia, PA) and HKCs were isolated from keratoconus corneas from Aarhus University Hospital, Aarhus, Denmark. Briefly, corneal epithelium and endothelium were removed from the stroma by scraping with a razor blade. The stromal tissue was cut into small pieces (2 × 2 mm) and placed into flasks with Eagle's Minimum Essential Medium (EMEM) with 10% fetal bovine serum (Biologicals: Lawrenceville, GA). Explants were allowed to adhere to the bottom of the plate. After 1–2 weeks of cultivation, the cells were passaged into a 100 mm cell culture plate. Cells were grown to confluency and seeded at concentrations of 10^6^ cells per well in 6-transwell plates prior to treatment. Cells were treated with Quercetin (3,3′,4′,5,7-pentahydroxyflavone) (Sigma Aldrich), lactic acid (Sigma Aldrich), and 2-o-alpha-D-glucopyranosyl-L-ascorbic acid (American Custom Chemicals Corporation). Quercetin was dissolved in DMSO. Less than 1% v/v of solvent was added to cell culture media. All other reagents were dissolved in ultrapure water unless otherwise stated. Controls were treated with vehicle. All reagents were filter-sterilized with a 0.2 micron filter prior to use in culture. Cells were treated for 2 or 4 weeks with cell lysis at the respective time point. The research adhered to the tenets of the Declaration of Helsinki.

### Protein extraction and western blot

We performed Western blot analysis from both the culture media and the seeded cells. Protein extraction from media following stimulation with lactate and/or Quercetin was pooled weekly by collecting the supernatant from each well. Maximal volume of sample was loaded in each lane and probed for Collagen I and III. For cell lysate protein extraction, lysates were extracted in RIPA buffer (50 mM Tris, pH 8, 150 mM NaCl, 1% Triton X-100, 0.1% SDS, 1% sodium deoxycholate) (Abcam; Cambridge, MA) supplemented with Complete Mini protease and phosphatase inhibitors (Sigma Aldrich; St. Louis, MO). Briefly, cell lysates were washed, lysed, centrifuged and stored at −20°C until further use. Total protein concentration was quantified using the Pierce BCA Protein Assay (Thermoscientific; Rockford, IL). Pre-cast sodium dodecyl sulfate (SDS)-polyacrylamide (PAGE) gels (Life technologies; Grand Island, NY) were loaded with 25μg of total protein. SDS-Polyacrylamide gel electrophoresis was performed on cell lysates under denaturing conditions. Western blot transfer was completed using nitrocellulose (Abcam; Cambridge; MA), blocked in 5% bovine serum albumin in TBST for 1 hour, and blots were probed overnight at 4°C in rabbit primary antibodies in 1% BSA: anti-Collagen I (ab34710; Abcam; Cambridge; MA), Collagen III (ab7778; Abcam; Cambridge; MA), Collagen V (ab94673; Abcam; Cambridge; MA), α-smooth muscle actin (ab5694; Abcam; Cambridge; MA), glyceraldehyde 3-phosphate dehydrogenase (GAPDH,ab9485; Abcam; Cambridge; MA) Secondary antibody Goat anti-Rb Alexafluor 568 (Life technologies; Grand Island; NY) was incubated with blot at room temperature for 1 hour with rocking. Quantification of bands was performed with densitometry using OMEGA Carestream MI Standard Program. All graphs depict Relative Expression (% Control) and error bars represent standard error of the mean (SEM).

### Metabolite extraction

Metabolites were isolated from cells as previously described^17^. Briefly, cells were washed with 1xPBS and lysed with ice-cold 80% methanol, incubated on dry ice for 15 minutes, and homogenized briefly to ensure complete cell lysis. Samples were then centrifuged (14,000 g, overnight, 4°C) and metabolites were isolated and stored at −80°C until use.

### Mass spectrometry

Samples were re-suspended in 20 μL HPLC-graded water for targeted tandem mass spectrometry (LC-MS/MS) and 5 μL were injected and analyzed using a hybrid 5500 QTRAP triple quadrupole mass spectrometer (AB/SCIEX) coupled to a Prominence UFLC system (Shimadzu) using an Amide HILIC column (Waters) and analyzed with selected reaction monitoring (SRM) with positive/negative polarity switching. Peak areas from the total ion current for each of 297 metabolite SRM transition were integrated using MultiQuant v2.1 software (AB/SCIEX)^23^. The resulting raw data from the MultiQuant software was uploaded to MetaboAnalyst (http://www.metaboanalyst.ca/MetaboAnalyst) for subsequent data processing and analyses^17^,^24^

### Statistical analysis

All experiments were repeated at least three times (separate donors) and data was analyzed for significant variation (p < 0.05) using two-way ANOVA and t-test where necessary (Graph Pad Prism 6 software).

## Author Contributions

T.M. and D.K. conceived and designed the experiments. T.M. performed the experiments and interpreted data. T.M., D.L., and A.S. prepared samples for metabolomics. S.P. assisted in cell culture. J.A. executed the metabolomics experiment. D.K. and T.M. wrote the manuscript.

## Figures and Tables

**Figure 1:**
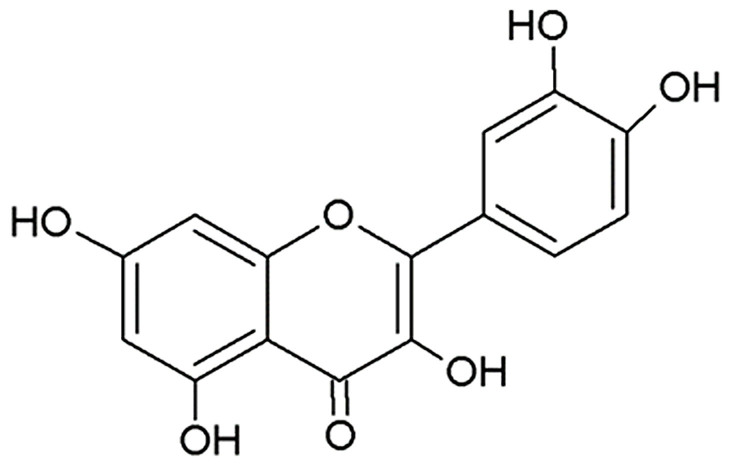
Chemical Structure of Quercetin (3,3′,4′,5,7-Pentahydroxyflavone) generated using Chem Sketch.

## Notes

A provisional patent application relating to the use of Quercetin for treating ocular conditions has been filed by the Board of Regents of the University of Oklahoma
